# DNA Methylation of Fibroblast Phenotypes and Contributions to Lung Fibrosis

**DOI:** 10.3390/cells10081977

**Published:** 2021-08-03

**Authors:** Poojitha Rajasekar, Jamie Patel, Rachel L. Clifford

**Affiliations:** Centre for Respiratory Research, Translational Medical Sciences, School of Medicine and NIHR Biomedical Research Centre, University of Nottingham, Nottingham NG7 2UH, UK; poojitha.rajasekar@nottingham.ac.uk (P.R.); Jamie.patel@nottingham.ac.uk (J.P.)

**Keywords:** DNA methylation, fibroblast, phenotype, fibrosis, lung

## Abstract

Fibroblasts are an integral part of connective tissue and play a crucial role in developing and modulating the structural framework of tissues by acting as the primary source of extracellular matrix (ECM). A precise definition of the fibroblast remains elusive. Lung fibroblasts orchestrate the assembly and turnover of ECM to facilitate gas exchange alongside performing immune functions including the secretion of bioactive molecules and antigen presentation. DNA methylation is the covalent attachment of a methyl group to primarily cytosines within DNA. DNA methylation contributes to diverse cellular phenotypes from the same underlying genetic sequence, with DNA methylation profiles providing a memory of cellular origin. The lung fibroblast population is increasingly viewed as heterogeneous with between 6 and 11 mesenchymal populations identified across health and lung disease to date. DNA methylation has been associated with different lung fibroblast populations in health and with alterations in lung disease, but to varying extents. In this review, we will discuss lung fibroblast heterogeneity and the evidence for a contribution from DNA methylation to defining cell populations and alterations in disease.

## 1. Introduction

Fibroblasts synthesize and integrate structural proteins including collagen and elastin into the extracellular matrix (ECM) of mesenchymal tissues [1]. They are an integral part of connective tissue and play a crucial role in developing and modulating the structural framework of tissues by acting as the primary source of ECM. Furthermore, they incorporate mechanical properties to the ECM while dynamically modulating the architecture [2]. Fibroblasts are proliferative and migratory in development but mainly quiescent and highly metabolically active in adult tissues [2]. Even though fibroblasts are prominent components of several organs, they were originally considered phenotypically and functionally homogenous [3]. They were defined by their spindle-shaped morphology and characterized by expression of vimentin, procollagen Iα2 and fibroblast specific protein-1 (FSP1) [1,4]. However, these markers are not fibroblast specific, and a precise definition of the fibroblast remains elusive. Increasingly fibroblast heterogeneity is recognized across developmental stages, tissue of origin and microenvironment [1], where they display an extensive variation in morphology, proliferation, function, molecular secretion (cytokines and ECM proteins) and molecular markers [4,5,6,7,8]. This heterogeneity is likely linked to their inherent plasticity and ability to specialize in different tissues [2]. Comparing transcriptomes of fibroblasts from the trachea, lung, abdomen, scalp, upper gingiva and soft palate [9] identified distinct gene expression profiles by body location. Furthermore, expression profiles of dermal fibroblasts from different anatomical locations [3,4], has identified functionally distinct fibroblast subtypes within a single tissue. Differences in transcriptional profiles likely reflect a combination of intrinsic differences (transcriptional regulation/epigenetics) and extrinsic factors including mechanical stress that differ between body regions. Overall fibroblasts subtype stratification is based on four factors: tissue state (i.e., healthy, disease, ageing, development, etc.), anatomic location based tissue composition and function (i.e., proportion of vascular, muscle or fat tissue), developmental origin and immediate microenvironment (i.e., ECM stiffness and cell-cell signaling) and finally cellular state (i.e., proliferative, migratory, differentiation, senescent, etc.) [2].

**Figure 1:**
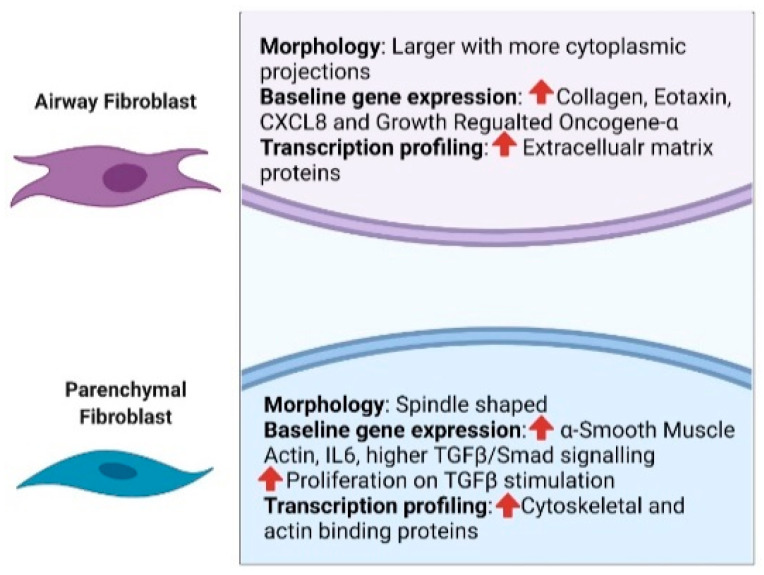
Distinction between airway and parenchymal fibroblasts based on morphology, baseline expression of distinct genes and pathways associated with transcription profile. Created with BioRender.com, accessed 25 July 2021.

The role of fibroblasts in human lung is multifaceted. Lung fibroblasts orchestrate the assembly and turnover of ECM to facilitate gas exchange alongside performing immune functions such as secretion of bioactive molecules and antigen presentation. Lung fibroblast heterogeneity is at the beginning of its investigation. However, studies comparing fibroblasts isolated from the airway and the parenchyma, and more recently single cell gene expression studies in whole lung sections have identified clear heterogeneity. Early studies clearly show fibroblasts isolated from the airways are different to parenchymal fibroblasts from distal parts of the lung. Airway fibroblasts (AFs) are larger with more cytoplasmic projections as opposed to the spindle shaped morphology of parenchymal fibroblasts (PFs). While AFs produce more collagen, eotaxin-1, CXCL8 and GRO-α at baseline [10], PFs express higher levels of α-smooth muscle actin and IL-6 [10] and are more proliferative on TGFβ stimulation [11]. Furthermore, PFs exhibit augmented TGF-β/Smad signaling at baseline compared to AFs [12]. Full transcriptome profiling between AFs and PFs identified more myofibroblast-like characteristics of PFs relative to AFs, via heightened SMAD3 activation (ratio of phosphor-SMAD3/total SMAD3) [13]. This study also showed distinct pathway associations of AFs and PFs; upregulation of ECM proteins was observed in AFs while cytoskeletal organization and actin binding proteins were upregulated in PFs [13] (Figure 1).

**Figure 2:**
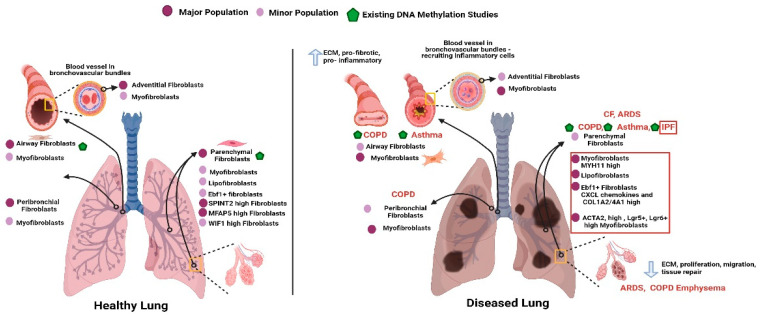
Fibroblast subtypes in healthy and diseased lungs. Diseased lung represents diseases associated with different regions of lung. The major and minor populations of fibroblasts in different pulmonary regions of healthy and diseased lungs are depicted along with indication of existing DNA methylation evidence corresponding to fibroblast subtype and pulmonary fibrotic phenotypes of common lung diseases. Created with BioRender.com, accessed 29 May 2021.

More recently, single cell RNA sequencing technology has facilitated discovery of new cellular phenotypes using cell type specific transcriptome signatures in tissues with heterogenous cell populations [14,15,16,17]. However, the nature of this data, the process and timing of its generation has resulting in some confounding profiles will different signatures sometimes being attributed to the same cell. Initial profiling in lung parenchyma identified two major SPINT2 high and MFAP5 high populations and a further minor population of WIF1 high fibroblasts that exhibited contrasting profiles of ECM gene transcription, suggestive of distinct functionality [18] (Figure 2). Subsequently, fibroblasts with distinct gene expression profiles were defined at distinct anatomical locations in the lung; peribronchial cells in the conducting airway wall, adventitial fibroblasts around the bronchovascular bundles and alveolar fibroblasts embedded in alveolar regions of the lung [17]. However, across all single cell datasets, including healthy and diverse lung disease tissue, between 6 and 11 mesenchymal populations have been identified to date [16]. The fibroblast subpopulations are anticipated to play unique roles in organizing and maintaining unique structure specific to the lung region, in addition to regional specific response to insults/exposures. It is likely that specific fibroblast subtypes will play key roles in wound healing, fibrotic diseases, cancer stroma, and potentially tissue aging [1].

Transcriptional profiling has extended our knowledge of fibroblast heterogeneity and phenotypes dramatically. It is likely that further studies that integrate other ‘omic approaches will provide further dimensionality to the understanding of cell lineage and specific fibroblast roles in human development and disease [19]. In this review we focus on DNA methylation as a relatively stable component of transcriptional regulation, that can establish and stabilize cellular phenotype by maintaining gene expression states [20] and can be transmitted with high fidelity during DNA replication [20]. We review evidence of a role for DNA methylation in defining fibroblast phenotypes in healthy lung and the implication in fibrotic mechanisms of lung diseases.

## 2. DNA Methylation

**Figure 3:**
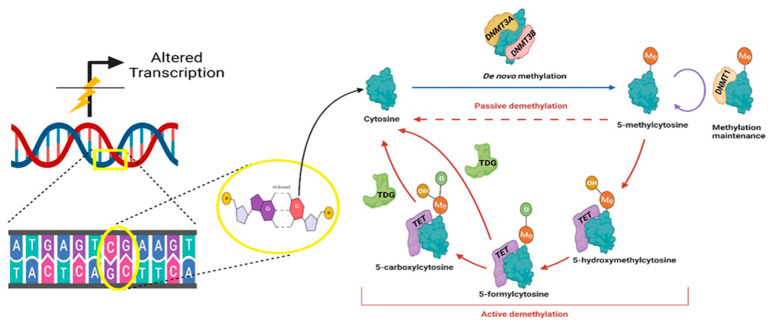
Mechanism of DNA methylation at position 5 of cytosines present within a CG dinucleotide site (CpG). Enzymes responsible for DNA methylation, passive demethylation and stepwise active demethylation processes are depicted (DNMT = DNA Methyl Transferases; TET = Ten-eleven Translocation enzymes; TDG = Thymine DNA Glycosylase). Created with BioRender.com, accessed 29 May 2021.

DNA methylation refers to the covalent attachment of a methyl group to DNA bases. DNA methylation on the fifth position of cytosine (5mC), is mainly restricted to CpG sites in vertebrates, with 60–80% of the ~29 million CpG sites in the human genome methylated [20,21]. The enzymes responsible for the methylation of cytosines are the DNA methyltransferases, DNMT1, DNMT3A, DNAMT3B and DNAMT3C [20]. Among these enzymes, DNMT3A and DNMT3B function as de novo methylation enzymes, while DNMT1 performs the role of maintaining DNA methylation signatures following hemi-methylation during replication [22,23] (Figure 3). DNA methylation can be removed via passive mechanisms involving loss of DNA methylation maintenance during rounds of replication, and active mechanisms utilizing the Ten-eleven translocation (TET) family of enzymes, TET1, TET2 and TET3 and Thymine DNA glycosylase (TDG) [24]. Promoter DNA methylation is a dynamic process that can transiently regulate gene expression in cell, tissue and disease specific manners by altering the transcription factor binding at gene promoters [25]. Cytosine methylation at genomic regions other than promoters such as gene body and intergenic regions also play gene regulatory roles but their relationship with gene expression remains complicated [26,27].

Techniques to measure DNA methylation levels have evolved extensively over the past two decades. Global DNA methylation profiling can be achieved by methylated DNA immunoprecipitation using a methyl cytosine antibody [28]. Genome wide DNA methylation can be profiled via whole genome bisulphite sequencing [29], reduced representation bisulphite sequencing (RRBS) [30] or array hybridization [31,32]. Each of these techniques relies on bisulfite treatment [33] of fragmented single stranded DNA to convert unmethylated cytosine molecules to uracil (which are subsequently converted to thymine during PCR amplification), leaving methylated cytosines and generating a sequence based difference from the original methylation difference. Whole genome bisulphite sequencing provides the densest coverage of DNA methylation, with RRBS providing higher resolution compared to probe hybridization arrays due to the limitation on the number of probes feasible within a microarray and difficultly of probe-based technologies distinguishing between repetitive genomic sequences. However, the ever-increasing coverage of the genome, ease of processing and relatively low cost of array-based platforms continues to make them the preferred technology for DNA methylation profiling at present, especially in large sample number studies [31]. For context, the very first array-based platform, the GoldenGate array scanned only 1536 CpGs across 371 genes and focused on cancer associated CpGs [34]. This was increased to 25,578 CpG sites mainly targeting CpG islands in gene promoters on the Illumina HumanMethylation27K [35]. The most recent Illumina bead chip array, HumanMethylationEPIC [36] profiles more than 850,000 CpG sites including >90% of the probes used in its predecessor the HumanMethylation450K Beadchip array [37]. Comprehensive high-throughput arrays for relative methylation (CHARM) is a further technology that combines tiling arrays and statistical procedures to improve specificity and sensitivity of the methylation profile at a CpG site by averaging information from adjacent genomic locations [38]. Targeted study of cytosine methylation at single nucleotide resolution can be achieved with pyrosequencing [39] and methylation specific PCR [40]. The most recent technological milestone in DNA methylation profiling is single cell DNA methylation analysis which will facilitate more accurate and in-depth investigations of cellular heterogeneity within a tissue/anatomical location. Integration of this data with other single cell ‘omics will open several avenues for better understanding of cellular phenotypes and disease pathogenesis [41].

## 3. DNA Methylation and Fibroblast Heterogeneity

DNA methylation contributes to diverse cellular phenotypes from the same underlying genetic sequence [42] with DNA methylation profiles providing a memory of cellular origin [20]. DNA methylation profiles of tissue and cultured cells distinguish tissues including lung, brain, heart, kidney, pancreas, skeletal muscle, and placenta. Importantly organ specific clustering is maintained in cultured cells suggesting tissue/cell type specific DNA methylation profiles are stable, including in culture [43]. Tissue specific DNA methylation profiles are postulated to mark or even drive differences in gene expression at genes functionally relevant to the tissue for example, cardiac and smooth muscle contraction in heart [44]. Regional differences in CpG island shores have been shown to better distinguish tissue and cell types, than differences in CpG islands [45].

DNA methylation profiling of different fibroblast populations is limited compared to transcriptional profiling; however, these studies do show large variation in DNA methylation profiles between fibroblasts from distinct locations, suggesting a high-level contribution from DNA methylation to different fibroblast phenotypes. DNA methylation profiles of fibroblasts cultured from the scalp versus the dura (layer of connective tissue that surrounds the brain), which are morphologically identical, showed 22% of CpGs profiled (Illumina HumanMethylation450K array) were differentially methylated between the fibroblast types, with >50% of effect sizes greater than a 10% difference in DNA methylation [42]. Fibroblast sampling location represented the majority of variation within the dataset suggesting a strong DNA methylation mediated memory of original cell location. Furthermore, human dermal fibroblasts from different anatomical sites (ear, arm, leg, abdomen and breast) cluster based on DNA methylation profile by anatomical sites, further suggesting positional memory exists even after culture [46].

Specifically, to the lung we have shown that fibroblasts isolated from the airway and parenchyma, display very distinct DNA methylation profiles [47]. While only a small proportion of the CpG sites differentially methylated between airway and parenchymal fibroblasts were associated with a transcriptional difference, methylation of CpG sites distinguished airway from parenchymal fibroblasts more effectively than gene expression, suggesting CpG methylation may provide an improved mechanism for lung fibroblast definition than gene expression. At present DNA methylation as a definer of lung fibroblast phenotype is overshadowed by single cell expression studies and an increased complexity of single cell DNA methylation profiling, but has good potential in the coming years.

## 4. DNA Methylation and Fibrosis in Lung Disease

Fibrosis encompasses a cascade of molecular processes including inflammation, abnormal accumulation of interstitial ECM proteins, increased proliferation of fibroblasts and subsequent imbalance between ECM formation and degradation. It is a progressive pathological event that results in increasing margins of fibrotic mass causing dysfunction of tissue and organs. Fibrosis in the lung is a complex event involving cascades of paracrine signaling between fibroblasts, lung airway and parenchymal cells and inflammatory cells. It follows the mechanism of fibroblast mediated wound healing, but without complete resolution of injury. A site of lung injury often begins with damaged epithelium and basement membrane which triggers inflammation mediated activation of fibroblasts, secretion of proteases and matrix proteins and remodeled ECM [48,49]. Fibrosis occurs in multiple lung diseases which we will focus on in turn, providing a summary of fibroblast heterogeneity, known contribution of DNA methylation and areas where future studies are warranted to further our understanding.

### 4.1. Idiopathic Pulmonary Fibrosis

Idiopathic pulmonary fibrosis (IPF) is a progressive, fatal lung disease, characterized by excessive extracellular matrix deposition in the lung interstitium, destruction of the normal parenchymal structure and progressive loss of pulmonary function [50,51]. The traditional dogma states that repeated epithelial injury causes secretion of mediators that result in fibroblast proliferation and differentiation into myofibroblasts, which subsequently deposit excessive levels of ECM resulting in increased tissue stiffness. Non-resolvable fibrosis causes a vicious cycle of fibroblast activation. The IPF parenchyma is probably the most well profiled by single cell expression profiling of the diseased lung states, offering a vast amount of data on the transcriptional profile of different cell types [15,21,52,53]. In IPF some focus has been on epithelial cells, as the “initiator cell”, identifying sub-lineages of epithelial ATII cells, basal cells and an ECM-producing epithelial population [51]. However, of the mesenchymal cells, myofibroblasts have been a focus due to their normal role in helping form alveoli in development and restore tissue integrity after injury [51] but also their pathological role as the primary drivers of ECM deposition in fibrosis and as the IPF effector cell with fibroblast synthesizing capacity doubled with airway smooth muscle line contractile characteristics. The most recent single cell fibroblast study by Liu et al. [16] was a comprehensive study using both new and previously published data to try to standardize the definition of fibroblasts subtypes. They found in both the healthy and IPF human parenchyma up to eight mesenchymal populations, with consistent identification of lipofibroblasts, myofibroblasts, smooth muscle cells, pericytes, a population homologous to murine Ebf1+ fibroblasts, an intermediate fibroblast subtype and mesothelial cells (Figure 2). Comparing healthy to IPF lungs, in lipofibroblasts collagen and ECM related genes were among the most differentially expressed, myofibroblasts expressed myosin heavy chain genes (MHY11) in IPF tissue and increased expression of other traditionally exclusive smooth muscle cells genes while in pericytes and Ebf1 fibroblasts CXCL chemokine and ECM related genes (COL1A2/4A1) were differentially expressed. In summary the data determined all mesenchymal subtypes, not just myofibroblasts as traditionally described, contributed to excessive ECM production in IPF without trans-differentiation of fibroblast type (Figure 2).

As with the healthy lung, transcriptional profiling is more advanced than DNA methylation profiling, however, DNA methylation is altered in parenchymal lung tissue from individuals with IPF compared to controls. First shown in 2012, of >14,000 genes represented by on the Illumina HumanMethylation27 BeadChip, 870 genes were differentially methylated in IPF lung tissue compared to controls, 35 of which linked to differential expression of the annotated gene [54] including previously identified IPF associated genes including MMP7 and COL3A1. Subsequently, across 4.6 million CpGs profiled by CHARM, 2130 differently methylated regions were identified between IPF tissue and controls. A third of the regions were within 5 kb of a gene that was differentially expressed in IPF versus control tissue suggesting DNA methylation contributes to differential gene expression in IPF parenchyma. DNA methylation associated gene expression was enriched for IPF implicated pathways including Wnt/β-catenin and epithelial adherens junction signaling [55]. However, using whole lung tissue does not allow for identification of the cell types in which the differential DNA methylation is occurring and, perhaps more importantly, due to the cell type specific nature of DNA methylation, IPF versus control differences could be driven by distinct cell composition of IPF lung. Indeed, Sanders et al., showed via lung tissue immunohistological staining of tissue sections matched to those in which DNA methylation data was generated, that increased DNMT3a staining, thought to be driving the changes to DNA methylation in IPF lung tissue, was primarily in epithelial cells overlying fibroblastic foci, indicating DNA methylation alteration may be primarily epithelial [54]. However, isolated parenchymal fibroblast DNA methylation has been profiled at low density (HumanMethylation 27 array) and small numbers (6 IPF patients, 3 non-fibrotic control patients and 3 normal lung fibroblast cell lines), identifying 125 differently methylated CpGs [56] in IPF versus control fibroblasts, with targeted analysis linking altered DNA methylation with changed gene expression (Figure 2). This highlights the potential for fibroblast specific aberrant DNA methylation in IPF that would benefit from further analysis on higher density scale in more donor samples. Even though considered preliminary as undertaken in a single cell line, parenchymal fibroblast DNA methylation can also be regulated by TGFβ stimulation, with a greater number of modifications in cells from an individual with IPF than from a healthy donor. This is potentially driven by increased TGFβ induced DNMT3a expression in IPF fibroblasts and suggests parenchymal fibroblasts from individuals with IPF may have a more plastic methylome in response to fibrotic stimuli and thus contribute to the vicious signaling cycle of IPF pathogenesis [57].

### 4.2. Asthma

Asthma is an inflammatory disease of the airway that manifests as bronchoconstriction, wheezing and shortness of breath. Structural changes within the asthmatic lung are driven by multiple cellular processes including epithelial cell damage and apoptosis, increased airway smooth muscle cell mass, aberrant and prolonged immune responses and fibroblast activation [58]. Genetic and environmental factors disrupt the homeostasis of the healthy lung which maintains levels of collagen and ECM proteins in equilibrium by regulation of synthesis and degradation, resulting in subepithelial fibrosis and thickening of the reticular basement membrane [59]. This process is primarily mediated by submucosal resident fibroblasts that are activated by TGFβ1, matrix metalloproteinases and tissue inhibitors of metalloproteinases [60,61,62], proliferate and differentiate into myofibroblasts [58]. Circulating fibroblasts expressing collagen I and CD34 are also recruited to asthmatic airways via chemokine and cytokine signaling and undergo transdifferentiation into myofibroblasts [63]. While dedifferentiation of airway smooth muscle cells into myofibroblasts occurs in subepithelial regions in close proximity to smooth muscle layer [64]. Fibroblast activation leads to deposition of ECM components collagen I, collagen III, collagen V fibronectin and tenascin [65] resulting in airway wall thickening, reduced airway distensibility and increased airflow limitation [59]. Fibrosis occurs early in asthma pathogenesis and is associated with severity of disease and resistance to therapy [59,66,67].

Functional alterations to sub-populations of fibroblasts in asthma have not been well investigated. Single cells expression studies have primarily focused on epithelial cell subpopulations and further focus on fibroblast populations has potential to vastly increase our understanding of fibrotic mechanisms in asthma. Genome wide gene expression comparison of airway versus parenchymal fibroblasts in asthma did not identify any differences between asthmatic and non-asthmatic donors in either fibroblast population however this was in relatively small donor numbers [13]. In contrast, we identified that differences in regional DNA methylation profiles associate with asthmatic status in both airway and parenchymal fibroblasts [47] (Figure 2). In this case, 17 and 112 differentially methylated DNA regions were identified in airway and parenchymal fibroblasts respectively, with similar donor numbers in each comparison, and no overlap between the fibroblast populations, suggesting individual contributions of DNA methylation to distinct fibroblast populations in asthma pathology as well as the healthy lung. Genes annotated to the differentially methylated DNA regions did not display any associated differential gene expression under baseline conditions. However, it is feasible that DNA methylation acts as a “memory” and differential gene expression levels only become apparent upon “cell activation”, for example via an inflammatory/allergic response, or in response to an inhaled exposure. Studies of the effects of DNA methylation on temporal gene expression in response to stimulation are needed to more fully elucidate the impact of DNA methylation differences associated with disease on gene expression and cell function. Mechanisms of asthma pathology mainly focus on the airway and this study highlighted perturbations to the parenchyma, where increased myofibroblast presence and ECM deposition have been reported [68,69].

### 4.3. Chronic Obstructive Pulmonary Disease (COPD)

Chronic Obstructive Pulmonary Disease (COPD) is a heterogenous disease of the lung, clinically defined by airflow obstruction that is not reversible and caused by inhalation of noxious particles or gases primarily from cigarette smoke. COPD involves two seemingly opposing components; parenchymal lung destruction (emphysema) with a loss of ECM deposition and small airways disease with increased ECM deposition in small airway fibrosis (Figure 2). Emphysema is characterized by destruction of the alveolar walls and a reduction in elastic recoil. The initial damage in emphysema occurs to the epithelial cells upon exposure however subsequent lung integrity relies on parenchymal fibroblasts and deposition of ECM components. In small airways disease, airways are narrowed with thickening and distortion of the airway wall [70] contributed to by peribronchial fibrosis [71]. As with fibrosis in asthma, small airways disease in COPD is an early feature and is linked to progression [72]. Due to these opposing fibroblast mediated pathologies in the COPD airway and parenchyma, it has been considered for some time that different populations of fibroblast contribute to the two different features of COPD, however only limited profiling has been performed. Small airway fibroblasts in COPD are profibrotic (secrete collagens 1A1/3A1, MMP2 and MMP9), pro-inflammatory (increased CXCL8 secretion), senescent (elevated p21 and p16 expression) [70] and express reduced levels of antioxidants (Superoxide dismutase 2 and 3) [73,74]. Parenchymal fibroblasts in COPD display reduced proliferation [75], reduced capability to sustain tissue repair (increased PGE2 production and EP2/EP4 expression, reduced response to TGFβ) [76], reduced contractility [76,77], reduced migration to chemoattractants [76] and increased expression and secretion of CXCL8 and IL-6 [78]. Two recent single cell RNA sequencing studies in lung tissue obtained from individuals with severe COPD and healthy individuals identified a distinct epithelial subpopulation in the alveolar niche that expressed hedgehog interacting protein (HHIP) and a ciliated epithelial cell population in peripheral lung parenchyma that expressed fibrosis associated proteins, Insulin Like Growth Factor Binding Protein 5 (IGFBP5) and protein quaking (QKI) [79]. However, to date, no single cell gene expression profiling of fibroblasts in COPD has been undertaken and represents a gap in the communities understanding of COPD pathology.

There is evidence for a link between DNA methylation and COPD pathogenesis [80,81,82,83,84]. Studies in blood [80] identified differential methylation in association with the presence and severity of COPD with CpG annotated genes representing immune/inflammatory pathways, response to stress and external stimuli and wound healing/coagulation pathways. In whole lung tissue, DNA methylation profiles linked to Endothelial PAS Domain Protein 1 (EPAS1) as a key regulator of COPD disease severity [81], and identified CpGs with differential methylation levels between lung tissue of smokers and individuals with COPD [82,83]. Studies in blood have limited translation to lung pathology and as with studies on IPF, those in lung tissue are complicated by mixed cell population and do not provide the granularity of cell type/cell population alterations. In small airway epithelial cells aberrant global DNA methylation was identified between former smokers with and without COPD [84]. More recently, we have shown in cultured airway and parenchymal fibroblasts from individuals with and without COPD, that COPD associates with regional differences in DNA methylation in both cell populations [85] (Figure 2). A greater number of DNA regions associated with COPD status in airway fibroblasts than parenchymal fibroblasts, potentially implicating DNA methylation as making a greater contribution to airways pathology in COPD. DNA methylation associated gene expression was only found in parenchymal fibroblasts, however this was undertaken in a targeted manner using CpG annotation to determine gene association and full genome wide expression profiling and expression quantitative trait methylation (eQTM) analysis would further expand our understanding of fibroblast population function in COPD. As for studies in asthma, it is important to note that gene expression was only assessed at a single time point and is intrinsically sensitive to variation and stimulation. We also performed a secondary DNA methylation analysis in both airway and parenchymal fibroblasts, to assess CpG methylation variability in COPD as opposed to differential DNA methylation. Differential DNA methylation compares mean level DNA methylation between cases and controls while assessment of variability identifies individual sites displaying “epigenetic outliers” in heterogeneous populations [86]. Differential variability analysis identified 359 differentially variable CpG sites between COPD and non-COPD parenchymal fibroblasts but none in airway fibroblasts. Of the three genes associated with differential variable DNA methylation targeted gene expression analysis identified significant gene expression differences associated with COPD in two of the genes and a strong trend toward differential expression in the third. This higher “success rate” at identifying DNA methylation associated gene expression differences associated with COPD suggested differential variable methylation may be a preferable method for identifying DNA methylation regulated alterations in gene expressions in heterogeneous disease such as COPD and could be expanded to other lung pathologies. Together, these data suggest that while larger alterations to DNA methylation occur in association with COPD status in airway fibroblasts the link to steady state gene expression is more pronounced in parenchymal fibroblasts, indicating not only differential response of CpG methylation to disease status but also potentially differential mechanistic function of the CpG methylation alteration.

### 4.4. Acute Respiratory Distress Syndrome (ARDS)

Respiratory infections that cause an acute inflammatory response in lungs known as acute respiratory distress syndrome (ARDS) predominantly present with pulmonary hypoxia, excessive infiltration of immune cells, oedema and result in mild to severe respiratory failure [87,88]. Infiltration of neutrophils and increased chemotactic and mitogenic cytokine production are the first steps of ARDS pathogenesis, with acute increases in pro-inflammatory cytokines including IL-2, IL-4, TNFα, IL1-β, CXCL8 and IL-6 in bronchoalveolar lavage [89]. In addition, pro-fibrotic cytokines including thrombin, fibrin and tissue factor VII enter the ARDS injury site through the circulation and contribute to progression of fibrosis along with suppressed fibrinolytic proteins (antiplasmin and plasmin activator inhibitor) [90,91]. Activated fibroblasts in ARDS are responsible for both interstitial and intra alveolar fibrosis through secretion of ECM proteins, predominantly collagen type I and III that forms a dense irregular matrix [92]. Two studies link alterations in whole blood DNA methylation to ARDS. Szilagyi et al., utilized hypothesis driven targeted DNA methylation profiling of myosin light chain kinase to link differential methylation to ARDS and a further effect modification by ethnicity [93]. While Guo et al., identified two CpGs associated with inflammation (Prostaglandin D2) and fibrosis (Internal membrane ATPase) linked to 28-day ARDS mortality risk via whole blood DNA methylation profiling [94]. Despite a link between ARDS pathogenesis and a pro-fibrotic phenotype of heterogenous lung fibroblast populations, the contribution of DNA methylation is yet to be understood.

### 4.5. Cystic Fibrosis

Cystic fibrosis is a multi-organ associated, genetic mutation-based disorder. Cystic fibrosis transmembrane conductance regulator (CFTR) gene mutation is the cause for this life shortening disease that results in progressive airway destruction through chronic.

Inflammation along with pancreatic insufficiency, ion and water transport imbalance in organs and male infertility. Although it affects multiple organs, the main cause of mortality/morbidity in cystic fibrosis is poor prognosis of lung dysfunction [95,96]. Persistent cycles of infection in cystic fibrosis activate an immune response followed by fibroblast proliferation, accumulation of ECM and lung fibrosis, however this fibrogenesis is not well characterized [97,98]. The involvement of DNA methylation in the regulation of cystic fibrosis pathogenesis has only been performed in easily accessible human tissues. Targeted bisulphite sequencing of CFTR and 13 lung disease modifier genes in nasal epithelial cells and whole blood identified significant association between DNA methylation levels in three genes (Heme Oxygenase 1, Glutathione S-Transferase Mu 3, Endothelin Receptor Type A) and disease severity [99]. Genome wide profiling of nasal epithelium DNA methylation in 32 CF patients and 16 controls showed DNA methylation differences between mild and severe CF and subsequent association with lung function in 50 CpG sites [100]. DNA methylation profiling of bronchoalveolar lavage cells collected from CF patients compared to healthy controls established significant methylation differences in 109 CpG sites [101]. Finally, Pineau et al. identified a robust CF biomarker (cg11702988, ATPase Phospholipid Transporting 11A gene) from nasal epithelial cell genomewide DNA methylation profiling of 51 adult CF samples and 24 healthy controls. The biomarker was validated in sputum cells using pyrosequencing and can be used for stratifying high risk and disease severity in CF patients [102]. Improvement to in vitro models, including a recently developed in vitro 3D stromal model [98], and single cell technologies will hopefully improve knowledge of cystic fibrosis fibroblast pathology including the involvement of DNA methylation.

## 5. Conclusions and Future Perspectives

In this review we have highlighted and summarized the current evidence for the contribution of DNA methylation to the complex fibroblast heterogeneity in the healthy lung and in fibrosis associated with multiple lung diseases (Table 1).

The development of single cell expression technology has seen an explosion of transcriptional profiling data of the lung in health and in disease, with DNA methylation profiling taking a back seat due to the increased complexity of profiling DNA methylation at the single cell level. DNA methylation profiling by next generation sequencing requires bisulphite conversion to convert cytosine methylation information into sequence-based information. The increased physical manipulation generates issues with levels of DNA remaining for sequencing, while the conversion of unmethylated cytosine to uracils makes alignment of sequencing data more difficult than for expression profiling. However, there is strong evidence that DNA methylation is involved in determining cell phenotype and work from ourselves and others has shown that DNA methylation profiles are associated fibroblast population in both health and lung disease. Increased optimization of single cell technologies to profile DNA methylation has large potential to inform our understanding of fibroblast phenotype, function and alteration in disease. Even though, it is important to consider that even with the wealth of information coming from single cell profiling, it now needs to be integrated with positional techniques so that we can determine exactly where within the lung cells are positioned and modifications in disease are occurring.

However, even upon profiling and definition of these cell types, challenges still remain. Even though it may be possible to isolate these subpopulations, they may not maintain their phenotype in culture [2]. Outgrowth techniques from tissue likely select out more proliferative subtypes and removing cells from their complex environment and placing them on generic tissue culture plastic in isolation will alter their expression and transcriptional profile. The development of more complex in vitro models, in parallel to ex vivo profiling, is paramount to being able to molecularly manipulate the cells in a manner that facilitates understanding the function of the distinct cell populations in health and disease, and the potential to target specific populations or signaling profiles of a specific population for therapeutic benefit.

The association between DNA methylation and gene expression is complex. While gene expression provides a snapshot of the functional transcriptome at the time, DNA methylation can represent a cellular memory that does not link directly to simultaneous gene expression levels. Furthermore, methylation of CpG sites can trans-regulate expression of genes distant from the CpG. Using targeted DNA methylation editing, such as dCas9-Dnmt3a/Tet1 to understand the role of site-specific DNA methylation in gene expression and cell fate determination will be important [20]. In vitro models of disease are also likely to be necessary for understanding trajectory of disease and the contribution of specific fibroblast populations and DNA methylation/gene expression to that trajectory. Animal models offer some insight here, but for human tissue we generally only have healthy and established disease samples, making it difficult to understand “which came first” [2].

The bulk of lung fibroblast transcriptional profiling has been undertaken in IPF resulting in a focus on parenchymal fibroblasts. However, our work has highlighted DNA methylation differences between airway and parenchymal fibroblasts in both health and asthma/COPD. Asthma is primarily considered an airways disease so identifying modification to parenchymal populations indicates the potential for modifications outside of our historically standard lung regions and moving forwards, as techniques improve and become more mainstream, it will be beneficial for studies to consider sampling different areas of the lung in both health and diverse lung diseases.

In conclusion, our understanding of lung fibroblast heterogeneity has increased dramatically over the last 5 years. This is particularly true of transcriptional profiling, but also for DNA methylation although on a more granular level. Improvements in technology over the next few years will allow us to integrate DNA methylation and other ‘omics data to further understand cellular phenotypes and their molecular definition.

## Figures and Tables

**Table 1:** Summary table of lung disease DNA methylation studies discussed.

Disease	Findings	Cell/Tissue Type	Array Platform ^1^	Ref
IPF	870 differentially methylated genes including IPF linked genes COL3A1 and MMP7	Whole Lung Tissue (*n* = 12 IPF; 7 Controls)	Illumina 27 k	[54]
IPF	2130 differentially methylated regions	Whole Lung Tissue (*n* = 94 IPF; 67 Controls)	CHARM	[55]
IPF	125 differentially methylated CpGs	Parenchymal fibroblasts (*n* = 6 IPF; 3 non-fibrotic Controls; 3 normal lung fibroblast cell lines	Illumina 27 k	[56]
Asthma	17 and 112 differentially methylated regions in airway and parenchymal fibroblast respectively. TWIST1 identified as airway vs. parenchymal fibroblast marker	Airway fibroblasts (*n* = 9 Asthma; 8 Controls) and Parenchymal fibroblasts (*n* = 8 Asthma; 9 Controls)	Illumina EPIC	[47]
COPD	349 disease severity associated differentially methylated CpGs	Whole Blood (2 cohorts with *n* = 620 COPD; 325 Controls and 181 COPD; 109 Controls)	Illumina 27 k	[81]
COPD	Identified EPAS1 as key regulator of COPD	Whole Lung Tissue (*n* = 176 COPD; 76 Controls)	Nimblegen 2.1	[82]
COPD	280 and 10 differentially methylated CpGs in COPD compared to non-smokers and smokers respectively.	Lung Tissue (*n* = 8 non-smokers; 8 smokers with normal lung function; 8 COPD)	Illumina 450 k	[83]
COPD	535 differentially methylated CpGs	Whole Lung Tissue (*n* = 114 COPD; 46 Controls)	Illumina 450 k	[84]
COPD	1260 differentially methylated CpGs	Small airway epithelial cells (*n* = 15 COPD; 23 Controls)	Illumina 27 k	[85]
COPD	887 and 44 differentially methylated regions in airway and parenchymal fibroblasts from COPD patients; 359 differentially variable CpGs in COPD parenchymal fibroblasts	Airway fibroblasts (*n* = 7COPD; 8 Controls) and Parenchymal fibroblasts (*n* = 29 COPD; 17 Controls)	Illumina 450 k	[86]
ARDS	2 differentially methylated CpGs on myosin light chain kinase gene associated with ARDS	Whole Blood (*n* = 39 ARDS; 75 ICU Controls)	Illumina 450 k	[94]
ARDS	2 differentially methylated CpGs located within prostaglandin D2 receptor and integral membrane ATPase genes	Whole Blood (185 moderate-to-severe ARDS)	Illumina 450 k	[95]
CF	Methylation changes in 3 genes (Heme Oxygenase 1, Glutathione S-Transferase Mu 3, Endothelin Receptor Type A) associated with disease severity	Nasal epithelial cells and whole blood (*n* = 48 CF; 24 Controls)	Targeted sequencing for CFTR and 13 lung disease modifier genes	[100]
CF	Differential methylation at 50 CpGs correlated with lung function in CF patients	Nasal epithelial cells (*n* = 32 CF; 16 Controls)	Illumina 450 k	[101]
CF	109 differentially methylated CpGs	Bronchoalveolar Lavage (*n* = 4 CF; 4 Controls)	Illumina EPIC	[102]
CF	Differential methylation at CpG (cg11702988) showed negative correlation with disease severity. Validated in sputum as a biomarker.	Nasal epithelial cells (*n* = 51 CF; 24 Controls)	Illumina 450 k	[101]

^1^ Illumina 27 k = Illumina HumanMethylation27 BeadChip; CHARM = comprehensive high-throughput arrays for relative methylation; Illumina EPIC = Illumina HumanMethylationEPIC BeadChip; Nimblegen 2.1 = Nimblegen 2.1 MWhole-Genome Tiling array; Illumina 450 k = Illumina HumanMethylation450K BeadChip; IPF = Idiopathic pulmonary fibrosis; COPD = chromic obstructive pulmonary disease; ARDS = Acute respiratory distress syndrome; CF = cystic fibrosis.

## Data Availability

Not applicable.

## References

[1] Lynch M.D., Watt F.M. (2018). Fibroblast heterogeneity: Implications for human disease. J. Clin. Investig..

[2] Shaw T.J., Rognoni E. (2020). Dissecting Fibroblast Heterogeneity in Health and Fibrotic Disease. Curr. Rheumatol. Rep..

[3] LeBleu V.S., Neilson E.G. (2020). Origin and functional heterogeneity of fibroblasts. FASEB J..

[4] Rinn J.L., Bondre C., Gladstone H.B., Brown P.O., Chang H.Y. (2006). Anatomic demarcation by positional variation in fibroblast gene expression programs. PLoS Genet..

[5] Fries K.M., Blieden T., Looney R.J., Sempowski G.D., Silvera M.R., Willis R.A., Phipps R.P. (1994). Evidence of fibroblast heterogeneity and the role of fibroblast subpopulations in fibrosis. Clin. Immunol. Immunopathol..

[6] Jordana M., Schulman J., McSharry C., Irving L.B., Newhouse M.T., Jordana G., Gauldie J. (1988). Heterogeneous proliferative characteristics of human adult lung fibroblast lines and clonally derived fibroblasts from control and fibrotic tissue. Am. Rev. Respir. Dis..

[7] Ko S.D., Page R.C., Narayanan A.S. (1977). Fibroblast heterogeneity and prostaglandin regulation of subpopulations. Proc. Natl. Acad. Sci. USA.

[8] Derdak S., Penney D.P., Keng P., Felch M.E., Brown D., Phipps R.P. (1992). Differential collagen and fibronectin production by thy1+ and thy1- lung fibroblast subpopulations. Am. J. Physiol..

[9] Foote A.G., Wang Z., Kendziorski C., Thibeault S.L. (2019). Tissue specific human fibroblast differential expression based on RNAsequencing analysis. BMC Genom..

[10] Dessalle K., Narayanan V., Kyoh S., Mogas A., Halayko A.J., Nair P., Baglole C.J., Eidelman D.H., Ludwig M.S., Hamid Q. (2016). Human bronchial and parenchymal fibroblasts display differences in basal inflammatory phenotype and response to IL-17A. Clin. Exp. Allergy.

[11] Kotaru C., Schoonover K.J., Trudeau J.B., Huynh M.L., Zhou X., Hu H., Wenzel S.E. (2006). Regional fibroblast heterogeneity in the lung: Implications for remodeling. Am. J. Respir. Crit. Care Med..

[12] Pechkovsky D.V., Hackett T.L., An S.S., Shaheen F., Murray L.A., Knight D.A. (2010). Human Lung Parenchyma but Not Proximal Bronchi Produces Fibroblasts with Enhanced TGF-beta Signaling and alpha-SMA Expression. Am. J. Respir. Cell Mol. Biol..

[13] Zhou X.X., Wu W., Hu H.Z., Milosevic J., Konishi K., Kaminski N., Wenzel S.E. (2011). Genomic Differences Distinguish the Myofibroblast Phenotype of Distal Lung Fibroblasts from Airway Fibroblasts. Am. J. Respir. Cell Mol. Biol..

[14] Raredon M.S.B., Adams T.S., Suhail Y., Schupp J.C., Poli S., Neumark N., Leiby K.L., Greaney A.M., Yuan Y.F., Horien C. (2019). Single-cell connectomic analysis of adult mammalian lungs. Sci. Adv..

[15] Reyfman P.A., Walter J.M., Joshi N., Anekalla K.R., McQuattie-Pimentel A.C., Chiu S., Fernandez R., Akbarpour M., Chen C.I., Ren Z.Y. (2019). Single-Cell Transcriptomic Analysis of Human Lung Provides Insights into the Pathobiology of Pulmonary Fibrosis. Am. J. Respir. Crit. Care Med..

[16] Liu X., Rowan S., Liang C.J., Yao C., Deng N., Huang G., Xie T., Wang Y., Stripp B.R., Noble P.W. (2020). Definition and Signatures of Lung Fibroblast Populations in Development and Fibrosis in Mice and Men. Am. J. Respir. Crit. Care Med..

[17] Tsukui T., Sun K.H., Wetter J.B., Wilson-Kanamori J.R., Hazelwood L.A., Henderson N.C., Adams T.S., Schupp J.C., Poli S.D., Rosas I.O. (2020). Collagen-producing lung cell atlas identifies multiple subsets with distinct localization and relevance to fibrosis. Nat. Commun..

[18] Valenzi E., Bulik M., Tabib T., Morse C., Sembrat J., Bittar H.T., Rojas M., Lafyatis R. (2019). Single-cell analysis reveals fibroblast heterogeneity and myofibroblasts in systemic sclerosis-associated interstitial lung disease. Ann. Rheum. Dis..

[19] Sucre J.M.S., Hagood J. (2020). Single cell analysis of human lung development: Knowing what mesenchymal cells are and what they may be. Eur. Respir. J..

[20] Kim M., Costello J. (2017). DNA methylation: An epigenetic mark of cellular memory. Exp. Mol. Med..

[21] Chen P.Y., Feng S.H., Joo J.W.J., Jacobsen S.E., Pellegrini M. (2011). A comparative analysis of DNA methylation across human embryonic stem cell lines. Genome Biol..

[22] Lyko F. (2018). The DNA methyltransferase family: A versatile toolkit for epigenetic regulation. Nat. Rev. Genet..

[23] Okano M., Bell D.W., Haber D.A., Li E. (1999). DNA methyltransferases Dnmt3a and Dnmt3b are essential for de novo methylation and mammalian development. Cell.

[24] Wu X.J., Zhang Y. (2017). TET-mediated active DNA demethylation: Mechanism, function and beyond. Nat. Rev. Genet..

[25] Suzuki M.M., Bird A. (2008). DNA methylation landscapes: Provocative insights from epigenomics. Nat. Rev. Genet..

[26] Aran D., Toperoff G., Rosenberg M., Hellman A. (2011). Replication timing-related and gene body-specific methylation of active human genes. Hum. Mol. Genet..

[27] Zemach A., McDaniel I.E., Silva P., Zilberman D. (2010). Genome-Wide Evolutionary Analysis of Eukaryotic DNA Methylation. Science.

[28] Weber M., Davies J.J., Wittig D., Oakeley E.J., Haase M., Lam W.L., Schubeler D. (2005). Chromosome-wide and promoter-specific analyses identify sites of differential DNA methylation in normal and transformed human cells. Nat. Genet..

[29] Harris R.A., Wang T., Coarfa C., Nagarajan R.P., Hong C.B., Downey S.L., Johnson B.E., Fouse S.D., Delaney A., Zhao Y.J. (2010). Comparison of sequencing-based methods to profile DNA methylation and identification of monoallelic epigenetic modifications. Nat. Biotechnol..

[30] Meissner A., Gnirke A., Bell G.W., Ramsahoye B., Lander E.S., Jaenisch R. (2005). Reduced representation bisulfite sequencing for comparative high-resolution DNA methylation analysis. Nucleic Acids Res..

[31] Gupta R., Nagarajan A., Wajapeyee N. (2010). Advances in genome-wide DNA methylation analysis. BioTechniques.

[32] Bock C. (2012). Analysing and interpreting DNA methylation data. Nat. Rev. Genet..

[33] Li Y., Tollefsbol T.O. (2011). DNA methylation detection: Bisulfite genomic sequencing analysis. Methods Mol. Biol..

[34] Bibikova M., Lin Z.W., Zhou L.X., Chudin E., Garcia E.W., Wu B., Doucet D., Thomas N.J., Wang Y.H., Vollmer E. (2006). High-throughput DNA methylation profiling using universal bead arrays. Genome Res..

[35] Bibikova M., Le J., Barnes B., Saedinia-Melnyk S., Zhou L.X., Shen R., Gunderson K.L. (2009). Genome-wide DNA methylation profiling using Infinium (R) assay. Epigenomics.

[36] Pidsley R., Zotenko E., Peters T.J., Lawrence M.G., Risbridger G.P., Molloy P., Van Djik S., Muhlhausler B., Stirzaker C., Clark S.J. (2016). Critical evaluation of the Illumina MethylationEPIC BeadChip microarray for whole-genome DNA methylation profiling. Genome Biol..

[37] Bibikova M., Barnes B., Tsan C., Ho V., Klotzle B., Le J.M., Delano D., Zhang L., Schroth G.P., Gunderson K.L. (2011). High density DNA methylation array with single CpG site resolution. Genomics.

[38] Irizarry R.A., Ladd-Acosta C., Carvalho B., Wu H., Brandenburg S.A., Jeddeloh J.A., Wen B., Feinberg A.P. (2008). Comprehensive high-throughput arrays for relative methylation (CHARM). Genome Res..

[39] Dupont J.M., Tost J., Jammes H., Gut N.G. (2004). De novo quantitative bisulfite sequencing using the pyrosequencing technology. Anal. Biochem..

[40] Sestakova S., Salek C., Remesova H. (2019). DNA Methylation Validation Methods: A Coherent Review with Practical Comparison. Biol. Proced. Online.

[41] Karemaker I.D., Vermeulen M. (2018). Single-Cell DNA Methylation Profiling: Technologies and Biological Applications. Trends Biotechnol..

[42] Ivanov N.A., Tao R., Chenoweth J.G., Brandtjen A., Mighdoll M.I., Genova J.D., McKay R.D., Jia Y., Weinberger D.R., Kleinman J.E. (2016). Strong Components of Epigenetic Memory in Cultured Human Fibroblasts Related to Site of Origin and Donor Age. PLoS Genet..

[43] Varley K.E., Gertz J., Bowling K.M., Parker S.L., Reddy T.E., Pauli-Behn F., Cross M.K., Williams B.A., Stamatoyannopoulos J.A., Crawford G.E. (2013). Dynamic DNA methylation across diverse human cell lines and tissues. Genome Res..

[44] Blake L.E., Roux J., Hernando-Herraez I., Banovich N.E., Perez R.G., Hsiao C.J., Eres I., Cuevas C., Marques-Bonet T., Gilad Y. (2020). A comparison of gene expression and DNA methylation patterns across tissues and species. Genome Res..

[45] Irizarry R.A., Ladd-Acosta C., Wen B., Wu Z., Montano C., Onyango P., Cui H., Gabo K., Rongione M., Webster M. (2009). The human colon cancer methylome shows similar hypo- and hypermethylation at conserved tissue-specific CpG island shores. Nat. Genet..

[46] Koch C.M., Suschek C.V., Lin Q., Bork S., Goergens M., Joussen S., Pallua N., Ho A.D., Zenke M., Wagner W. (2011). Specific age-associated DNA methylation changes in human dermal fibroblasts. PLoS ONE.

[47] Clifford R.L., Yang C.X., Fishbane N., Patel J., MacIsaac J.L., McEwen L.M., May S.T., Castellanos-Uribe M., Nair P., Obeidat M. (2020). TWIST1 DNA methylation is a cell marker of airway and parenchymal lung fibroblasts that are differentially methylated in asthma. Clin. Epigenet..

[48] Selman M., Pardo A. (2001). Idiopathic pulmonary fibrosis: An epithelial/fibroblastic cross-talk disorder. Respir. Res..

[49] Brody A.R., Soler P., Basset F., Haschek W.M., Witschi H. (1981). Epithelial-mesenchymal associations of cells in human pulmonary fibrosis and in bht-oxygen-induced fibrosis in mice. Exp. Lung Res..

[50] John A.E., Joseph C., Jenkins G., Tatler A.L. (2021). COVID-19 and pulmonary fibrosis: A potential role for lung epithelial cells and fibroblasts. Immunol. Rev..

[51] Nemeth J., Schundner A., Frick M. (2020). Insights Into Development and Progression of Idiopathic Pulmonary Fibrosis From Single Cell RNA Studies. Front. Med..

[52] Adams T.S., Schupp J.C., Poli S., Ayaub E.A., Neumark N., Ahangari F., Chu S.G., Raby B.A., DeIuliis G., Januszyk M. (2020). Single-cell RNA-seq reveals ectopic and aberrant lung-resident cell populations in idiopathic pulmonary fibrosis. Sci. Adv..

[53] Habermann A.C., Gutierrez A.J., Bui L.T., Yahn S.L., Winters N.I., Calvi C.L., Peter L., Chung M.I., Taylor C.J., Jetter C. (2020). Single-cell RNA sequencing reveals profibrotic roles of distinct epithelial and mesenchymal lineages in pulmonary fibrosis. Sci. Adv..

[54] Sanders Y.Y., Ambalavanan N., Halloran B., Zhang X., Liu H., Crossman D.K., Bray M., Zhang K., Thannickal V.J., Hagood J.S. (2012). Altered DNA methylation profile in idiopathic pulmonary fibrosis. Am. J. Respir. Crit. Care Med..

[55] Yang I.V., Pedersen B.S., Rabinovich E., Hennessy C.E., Davidson E.J., Murphy E., Guardela B.J., Tedrow J.R., Zhang Y., Singh M.K. (2014). Relationship of DNA methylation and gene expression in idiopathic pulmonary fibrosis. Am. J. Respir. Crit. Care Med..

[56] Huang S.K., Scruggs A.M., McEachin R.C., White E.S., Peters-Golden M. (2014). Lung fibroblasts from patients with idiopathic pulmonary fibrosis exhibit genome-wide differences in DNA methylation compared to fibroblasts from nonfibrotic lung. PLoS ONE.

[57] Negreros M., Hagood J.S., Espinoza C.R., Balderas-Martinez Y.I., Selman M., Pardo A. (2019). Transforming growth factor beta 1 induces methylation changes in lung fibroblasts. PLoS ONE.

[58] Hough K.P., Curtiss M.L., Blain T.J., Liu R.M., Trevor J., Deshane J.S., Thannickal V.J. (2020). Airway Remodeling in Asthma. Front. Med..

[59] Royce S.G., Cheng V., Samuel C.S., Tang M.L. (2012). The regulation of fibrosis in airway remodeling in asthma. Mol. Cell. Endocrinol..

[60] Mautino G., Henriquet C., Gougat C., Le Cam A., Dayer J.M., Bousquet J., Capony F. (1999). Increased expression of tissue inhibitor of metalloproteinase-1 and loss of correlation with matrix metalloproteinase-9 by macrophages in asthma. Lab. Investig..

[61] Vignola A.M., Riccobono L., Mirabella A., Profita M., Chanez P., Bellia V., Mautino G., D’Accardi P., Bousquet J., Bonsignore G. (1998). Sputum metalloproteinase-9 tissue inhibitor of metalloproteinase-1 ratio correlates with airflow obstruction in asthma and chronic bronchitis. Am. J. Respir. Crit. Care Med..

[62] Hayakawa T., Yamashita K., Tanzawa K., Uchijima E., Iwata K. (1992). Growth-promoting activity of tissue inhibitor of metalloproteinases-1 (timp-1) for a wide-range of cells—A possible new growth-factor in serum. FEBS Lett..

[63] Schmidt M., Sun G., Stacey M.A., Mori L., Mattoli S. (2003). Identification of circulating fibrocytes as precursors of bronchial myofibroblasts in asthma. J. Immunol..

[64] Gizycki M.J., Adelroth E., Rogers A.V., Obyrne P.M., Jeffery P.K. (1997). Myofibroblast involvement in the allergen-induced late response in mild atopic asthma. Am. J. Respir. Cell Mol. Biol..

[65] Postma D.S., Timens W. (2006). Remodeling in asthma and chronic obstructive pulmonary disease. Proc. Am. Thorac. Soc..

[66] Minshall E.M., Leung D.Y.M., Martin R.J., Song Y.L., Cameron L., Ernst P., Hamid Q. (1997). Eosinophil-associated TGF-beta(1) mRNA expression and airways fibrosis in bronchial asthma. Am. J. Respir. Cell Mol. Biol..

[67] Chetta A., Foresi A., DelDonno M., Bertorelli G., Pesci A., Olivieri D. (1997). Airways remodeling is a distinctive feature of asthma and is related to severity of disease. Chest.

[68] Boser S.R., Mauad T., Araujo-Paulino B.B., Mitchell I., Shrestha G., Chiu A., Butt J., Kelly M.M., Caldini E., James A. (2017). Myofibroblasts are increased in the lung parenchyma in asthma. PLoS ONE.

[69] Weitoft M., Andersson C., Andersson-Sjoland A., Tufvesson E., Bjermer L., Erjefalt J., Westergren-Thorsson G. (2014). Controlled and uncontrolled asthma display distinct alveolar tissue matrix compositions. Respir. Res..

[70] Barnes P.J. (2019). Small airway fibrosis in COPD. Int. J. Biochem. Cell Biol..

[71] Jeffery P.K. (2004). Remodeling and inflammation of bronchi in asthma and chronic obstructive pulmonary disease. Proc. Am. Thorac. Soc..

[72] Hogg J.C., Chu F., Utokaparch S., Woods R., Elliott W.M., Buzatu L., Cherniack R.M., Rogers R.M., Sciurba F.C., Coxson H.O. (2004). The nature of small-airway obstruction in chronic obstructive pulmonary disease. N. Engl. J. Med..

[73] Wrench C.L., Baker J.R., Fenwick P.S., Donnelly L.E., Barnes P.J. (2019). Chylous cardiac tapenade with chylothoracies secondary to Hodgkin’s lymphoma: The first recorded case of successful octreotide treatment. D42. Pleural Disease Case Reports II.

[74] Wrench C., Baker J., Fenwick P., Donnelly L., Barnes P. (2018). Small airway fibroblasts from COPD patients are senescent and pro-fibrotic. Eur. Respir. J..

[75] Holz O., Zuhlke I., Jaksztat E., Muller K.C., Welker L., Nakashima M., Diemel K.D., Branscheid D., Magnussen H., Jorres R.A. (2004). Lung fibroblasts from patients with emphysema show a reduced proliferation rate in culture. Eur. Respir. J..

[76] Togo S., Holz O., Liu X., Sugiura H., Kamio K., Wang X., Kawasaki S., Ahn Y., Fredriksson K., Skold C.M. (2008). Lung fibroblast repair functions in patients with chronic obstructive pulmonary disease are altered by multiple mechanisms. Am. J. Respir. Crit. Care Med..

[77] Campbell J.D., McDonough J.E., Zeskind J.E., Hackett T.L., Pechkovsky D.V., Brandsma C.A., Suzuki M., Gosselink J.V., Liu G., Alekseyev Y.O. (2012). A gene expression signature of emphysema-related lung destruction and its reversal by the tripeptide GHK. Genome Med..

[78] Zhang J., Wu L., Qu J.M., Bai C.X., Merrilees M.J., Black P.N. (2012). Pro-inflammatory phenotype of COPD fibroblasts not compatible with repair in COPD lung. J. Cell Mol. Med..

[79] Li X.Y., Noell G., Tabib T., Gregory A.D., Bittar H.T.E., Vats R., Kaminski T.W., Sembrat J., Snyder M.E., Chandra D. (2021). Single cell RNA sequencing identifies IGFBP5 and QKI as ciliated epithelial cell genes associated with severe COPD. Respir. Res..

[80] Qiu W., Baccarelli A., Carey V.J., Boutaoui N., Bacherman H., Klanderman B., Rennard S., Agusti A., Anderson W., Lomas D.A. (2012). Variable DNA methylation is associated with chronic obstructive pulmonary disease and lung function. Am. J. Respir. Crit. Care Med..

[81] Yoo S., Takikawa S., Geraghty P., Argmann C., Campbell J., Lin L., Huang T., Tu Z., Foronjy R.F., Spira A. (2015). Integrative analysis of DNA methylation and gene expression data identifies EPAS1 as a key regulator of COPD. PLoS Genet..

[82] Sundar I.K., Yin Q., Baier B.S., Yan L., Mazur W., Li D., Susiarjo M., Rahman I. (2017). DNA methylation profiling in peripheral lung tissues of smokers and patients with COPD. Clin. Epigenet..

[83] Morrow J.D., Cho M.H., Hersh C.P., Pinto-Plata V., Celli B., Marchetti N., Criner G., Bueno R., Washko G., Glass K. (2016). DNA methylation profiling in human lung tissue identifies genes associated with COPD. Epigenetics.

[84] Vucic E.A., Chari R., Thu K.L., Wilson I.M., Cotton A.M., Kennett J.Y., Zhang M., Lonergan K.M., Steiling K., Brown C.J. (2014). DNA methylation is globally disrupted and associated with expression changes in chronic obstructive pulmonary disease small airways. Am. J. Respir. Cell Mol. Biol..

[85] Clifford R.L., Fishbane N., Patel J., MacIsaac J.L., McEwen L.M., Fisher A.J., Brandsma C.A., Nair P., Kobor M.S., Hackett T.L. (2018). Altered DNA methylation is associated with aberrant gene expression in parenchymal but not airway fibroblasts isolated from individuals with COPD. Clin. Epigenet..

[86] Paul D.S., Teschendorff A.E., Dang M.A., Lowe R., Hawa M.I., Ecker S., Beyan H., Cunningham S., Fouts A.R., Ramelius A. (2016). Increased DNA methylation variability in type 1 diabetes across three immune effector cell types. Nat. Commun..

[87] Matthay M.A., Zemans R.L., Zimmerman G.A., Arabi Y.M., Beitler J.R., Mercat A., Herridge M., Randolph A.G., Calfee C.S. (2019). Acute respiratory distress syndrome. Nat. Rev. Dis. Primers.

[88] Soares M.P., Teixeira L., Moita L.F. (2017). Disease tolerance and immunity in host protection against infection. Nat. Rev. Immunol..

[89] Donnelly S.C., Haslett C. (1992). Cellular mechanisms of acute lung injury—Implications for future treatment in the adult respiratory-distress syndrome. Thorax.

[90] Dawes K.E., Gray A.J., Laurent G.J. (1993). Thrombin stimulates fibroblast chemotaxis and replication. Eur. J. Cell Biol..

[91] Bachofen M., Weibel E.R. (1982). Structural alterations of lung parenchyma in the adult respiratory-distress syndrome. Clin. Chest Med..

[92] Marshall R., Bellingan G., Laurent G. (1998). The acute respiratory distress syndrome: Fibrosis in the fast lane. Thorax.

[93] Szilagyi K.L., Lw C., Zhang X., Wang T., Fortman J.D., Zhang W., Garcia J.G.N. (2017). Epigenetic contribution of the myosin light chain kinase gene to the risk for acute respiratory distress syndrome. Transl. Res..

[94] Guo Y.C., Zhang R.Y., Zhu Z.Z., Shen S.P., Su L., Christiani D.C. (2018). Epigenome-wide association study for 28-day survival of acute respiratory distress syndrome. Intens. Care Med..

[95] Davies J.C., Alton E., Bush A. (2007). Cystic fibrosis. BMJ Br. Med. J..

[96] Cutting G.R. (2015). Cystic fibrosis genetics: From molecular understanding to clinical application. Nat. Rev. Genet..

[97] Rout-Pitt N., Farrow N., Parsons D., Donnelley M. (2018). Epithelial mesenchymal transition (EMT): A universal process in lung diseases with implications for cystic fibrosis pathophysiology. Respir. Res..

[98] Mazio C., Scognamiglio L.S., De Cegli R., Galietta L.J.V., Di Bernardo D., Casale C., Urciuolo F., Imparato G., Netti P.A. (2020). Intrinsic Abnormalities of Cystic Fibrosis Airway Connective Tissue Revealed by an In Vitro 3D Stromal Model. Cells.

[99] Magalhaes M., Rivals I., Claustres M., Varilh J., Thomasset M., Bergougnoux A., Mely L., Leroy S., Corvol H., Guillot L. (2017). DNA methylation at modifier genes of lung disease severity is altered in cystic fibrosis. Clin. Epigenet..

[100] Magalhaes M., Tost J., Pineau F., Rivals I., Busato F., Alary N., Mely L., Leroy S., Murris M., Caimmi D. (2018). Dynamic changes of DNA methylation and lung disease in cystic fibrosis: Lessons from a monogenic disease. Epigenomics.

[101] Chen Y.D.H., Armstrong D.A., Salas L.A., Hazlett H.F., Nymon A.B., Dessaint J.A., Aridgides D.S., Mellinger D.L., Liu X.Y., Christensen B.C. (2018). Genome-wide DNA methylation profiling shows a distinct epigenetic signature associated with lung macrophages in cystic fibrosis. Clin. Epigenet..

[102] Pineau F., Caimmi D., Taviaux S., Reveil M., Brosseau L., Rivals I., Drevait M., Vachier I., Claustres M., Chiron R. (2021). DNA Methylation at ATP11A cg11702988 Is a Biomarker of Lung Disease Severity in Cystic Fibrosis: A Longitudinal Study. Genes.

